# What Are the Benefits of Pet Ownership and Care Among People With Mild-to-Moderate Dementia? Findings From the IDEAL programme

**DOI:** 10.1177/0733464820962619

**Published:** 2020-10-07

**Authors:** Carol Opdebeeck, Michael A. Katsaris, Anthony Martyr, Ruth A. Lamont, James A. Pickett, Isla Rippon, Jeanette M. Thom, Christina Victor, Linda Clare

**Affiliations:** 1 Manchester Metropolitan University, UK; 2 University of Exeter, UK; 3 Alzheimer’s Society, London, UK; 4 Brunel University London, Uxbridge, UK; 5 University New South Wales Sydney, Australia

**Keywords:** quality of life, Alzheimer’s disease, loneliness, depression, animals/pets

## Abstract

Pet ownership has been associated with positive outcomes in many populations, yet the associations with physical and psychological wellbeing in people with dementia remain unclear. The current study used baseline data from 1,542 people living at home with mild-to-moderate dementia from the Improving the experience of Dementia and Enhancing Active Life (IDEAL) programme. Regression analyses investigated associations of pet ownership and pet care with self-reports of walking, loneliness, depression, and quality of life (QoL). After adjusting for covariates, having any pet was associated with higher likelihood of walking over 3 hr in the last week. Those with a dog and who were involved in its care were less likely to be lonely than those with no dog. Having any pet but no involvement in its care was associated with increased depression and decreased QoL compared with those without a pet. The key factor in the associations was involvement in the care of the pet by the person with dementia.

## Introduction

In the United Kingdom there are approximately 850,000 people living with dementia, around two thirds of whom reside at home (Prince et al., 2014). In the United States, approximately 80% of the 5.5 million people living with dementia reside at home (Lepore et al., 2017). As dementia prevention or cure remains elusive, a key policy and research focus has been on helping those with dementia to live well (Clare et al., 2019; Department of Health, 2015; Martyr et al., 2018). Part of living well with dementia is being able to continue activities that were enjoyed prior to diagnosis (Harding et al., 2019). Pet ownership may be one such activity and is common among the general population (Obradović et al., 2019; People’s Dispensary for Sick Animals [PDSA], 2019).

Within the general U.K. adult population, 49% of people own a pet (PDSA, 2019). It has been argued that pet ownership could promote positive psychological outcomes such as reduced loneliness, decreased stress following the loss of a loved one, and depression, through companionship and purpose, as well as improving physical health through the increased activity required to look after pets (Barker & Wolen, 2008; Beetz et al., 2012; Curl et al., 2016; Dall et al., 2017; Hughes et al., 2020; Hui Gan et al., 2019; Janevic et al., 2019; Krause-Parello, 2012; Obradović et al., 2019). Not all research has supported this proposition. Some studies have reported more equivocal results, for example, higher levels of depressive symptoms and loneliness in pet owners (Gee & Mueller, 2019; Obradović et al., 2019; Parslow et al., 2005; Pikhartova et al., 2014) potentially because people get a pet in response to loneliness or depression (Pikhartova et al., 2014). It should also be noted that the majority of this research is from Western cultures, and the results may be conflicting as some of the associations may be bidirectional. For instance, a large longitudinal cohort study identified that loneliness at earlier time points was associated with pet ownership, in addition to pet ownership being associated with future reports of loneliness in women (Pikhartova et al., 2014).

It is unclear from existing research how pet ownership may affect those living with dementia. For those with cognitive impairments, pets may provide nonjudgemental interactions that are reassuring, but pet ownership or pet care may also place a burden on the person with dementia. Identifying the benefits and difficulties of pet ownership in people living with dementia could help when making difficult decisions around pet ownership and inform future interventions. The majority of research in this area involving people with dementia has examined Animal-Assisted Interventions (AAI). AAI are interventions that are characterized by an interaction with a trained animal, generally in a controlled environment (Nordgren & Engstrom, 2014; Pitheckoff et al., 2018). For example, a trained dog may be brought into a nursing home and participants in the AAI provided with a time-limited opportunity to engage with the animal via petting, brushing or giving the animal treats. There is limited evidence that AAI can benefit people with dementia, albeit of varying quality. AAI have been associated with reduced depression and agitation and increased social interaction, cognitive function and quality of life (QoL; Freedman et al., 2020; Menna et al., 2016; Travers et al., 2013; Wong & Breheny, 2020; Yakimicki et al., 2019) but little to support the premise that AAI is beneficial for people with dementia (e.g., Wong & Breheny, 2020; Zafra-Tanaka et al., 2019). The experience of interacting with animals within residential or day care settings is likely to be fundamentally different from that experienced by pet owners living in the community. For example, pet owners are able to spend more time with and take greater ownership of the animal (Obradović et al., 2019), which may have implications for the ability to bond and interact with the animal.

We identified only one published study that sought to investigate the role of pets in the lives of people living with dementia as part of a larger American intervention study for female spouses of men with dementia. Connell et al. (2007) conducted telephone interviews with 62 female spouses of men with dementia who had a pet. Participants reported that pets played a unique support role, promoted calm, offered focus, diversion and distraction, and provided companionship or friendship for their spouse with dementia. However, they also reported that the relationship between the person with dementia and the pet had changed since diagnosis and the carers themselves had less time to care for the pets. To date no study has considered the association of pet ownership with physical activity, loneliness, depression, and QoL, or the extent to which the individual is directly involved in caring for the pet as opposed to simply living in the same household as the pet.

To summarize, previous research with wider society has shown that having a pet and being involved in caring for it is associated with greater physical activity, better cardiovascular health, less depression and loneliness, and better QoL (Barker & Wolen, 2008; Beetz et al., 2012; Curl et al., 2016; Dall et al., 2017; Hughes et al., 2020; Hui Gan et al., 2019; Janevic et al., 2019; Krause-Parello, 2012). Meanwhile, in relation to dementia the majority of research on the effects of interacting with animals has been conducted with people living in residential care where animals are used as part of an intervention; however, this is markedly different to owning and caring for a pet as a natural part of everyday life. The current study aimed to expand our understanding of the role pets can play in the lives of people with dementia by investigating the associations quantitatively within a large cohort of community-dwelling people with mild-to-moderate dementia. The study utilizes baseline data from the Improving the experience of Dementia and Enhancing Active Life (IDEAL) programme which asks a large cohort of people with dementia themselves about pet ownership, alongside other important outcome variables (Clare et al., 2014; Silarova et al., 2018). The overarching aim of the current study was to investigate whether having a pet, and the degree of involvement in its care, was associated with physical activity, loneliness, depression or QoL in people living with dementia. As most AAI studies in people with dementia have employed dogs, we wanted to investigate whether there was a stronger association with dog ownership compared with ownership of other animals in community-dwelling people with dementia.

## Method

### Design

The current study utilized baseline data from the IDEAL programme; a longitudinal cohort study of people with dementia and carers. Details of the aims and procedures can be found in the programme protocols (Clare et al., 2014; Silarova et al., 2018). The IDEAL study was approved by the Wales Research Ethics Committee 5 (reference 13/WA/0405) and the Ethics Committee of the School of Psychology, Bangor University (reference 2014-11684). IDEAL is registered with the U.K. Clinical Research Network, number 16593. The present study utilized IDEAL baseline data version 4.0.

### Study Population

At baseline the IDEAL cohort comprised 1,547 people with dementia and 1,283 carers. Our article focuses on the views of people with dementia. Inclusion criteria were a clinical diagnosis of dementia (any subtype) and a Mini-Mental State Examination (MMSE; Folstein et al., 1975) score of 15 or above (indicating mild-to-moderate stages of dementia), and participants had to be residing in the community at the time of enrolment into the study. Participant recruitment took place across 29 National Health Service (NHS) sites between July 2014 and August 2016. Exclusion criteria were terminal illness, inability to provide informed consent, and any known potential for home visits to pose a danger to researchers.

### Measures

Pet ownership was assessed through several questions. Participants were asked if they had no pets, one pet, or more than one pet. If they had a pet, they were asked to specify the type of animal(s); questions were adapted from Connell et al. (2007). As previous research has found involvement in caring for the animal to be an important factor (Parslow et al., 2005), a question asking whether the person with dementia was involved in the care of the animal was also included and responses were dichotomized into no involvement in care versus involvement in the care of the animal.

### Outcome Measures

Physical activity was assessed with a single question about how much walking the participant had done in the previous week, taken from the General Practice Physical Activity Questionnaire (GPPAQ; National Health Service, 2009). To match the U.K. NHS (2019) recommendations of at least 150 min of moderate activity per week, participants were grouped into those who walked less than 3 hr in the last week and those who had walked 3 hr or more in the last week.

Loneliness was assessed with the De Jong Gierveld 6-item loneliness scale (DJG-6; De Jong Gierveld & Van Tilburg, 2006). This scale comprises two subscales assessing social and emotional loneliness. The scales were combined to provide an overall loneliness score with possible scores ranging from zero to six with higher scores indicating greater loneliness. In accordance with the measure guidance and previous research, participants were allocated on the basis of their scores to one of three groups: not lonely (score of 0–1), moderately lonely (scores of 2–4), and severely lonely (scores of 5–6; De Jong Gierveld & Van Tilburg, 1999). As there was a very low number of participants in the severely lonely group (*n* = 75) in the IDEAL cohort (see Victor et al., 2020, for more detail), the severely and moderately lonely categories were grouped together into a “lonely” group, as in previous IDEAL programme studies (e.g., Clare et al., in press). The authors report good reliability (Cronbach’s α between .70 and .76, De Jong Gierveld & Van Tilburg, 2006). In the IDEAL cohort DJG-6 has a Cronbach’s alpha of .67.

The Geriatric Depression Scale-10 (GDS-10; Almeida & Almeida, 1999) was used to measure depression in participants living with dementia, with higher scores indicating more self-reported depressive symptoms. For the purposes of the analysis, the sample was dichotomized into not depressed (GDS-10 = 0–3) and depressed (GDS-10 = 4–10), as in previous IDEAL programme studies (e.g., Clare et al., in press; Wu et al., 2019). The measure has good reliability (Cronbach’s α = .75) and is a suitable screening instrument for major depression as defined by the Diagnostic and Statistical Manual of Mental Disorders (DSM-IV; 4th ed.; American Psychiatric Association, 1994) with a test–retest reliability of .84 (Almeida & Almeida, 1999). In the IDEAL cohort GDS-10 has a Cronbach’s alpha of .74.

The Quality of Life in Alzheimer’s Disease scale (QoL-AD; Logsdon et al., 2000) assessed QoL. The measure comprises 13 items with responses given on a 4-point scale (1 = *poor* to 4 = *excellent*) and incorporates multiple aspects of life including financial situation, relationships, health status, and mood. Scores were summed to provide a total ranging from 13 to 52 with higher scores indicating more positive ratings of QoL. The scale has good reliability (Cronbach’s α = .84 to .88) and good test–retest reliability (.76–.92; Logsdon et al., 2000). In the IDEAL cohort QoL-AD has a Cronbach’s alpha of .86.

### Covariates

Covariates considered in the analyses included key variables identified as having an association with the outcomes in previous IDEAL publications: age, gender, type of dementia—Alzheimer’s disease (AD) versus non-Alzheimer’s dementia (non-AD), functional ability, living situation (alone, with spouse/partner or with others; see Clare et al., in press, for details), and cognitive function. Functional ability was assessed with a modified 11-item Functional Abilities Questionnaire (FAQ; Martyr et al., 2012; Pfeffer et al., 1982) with participants grouped into six levels from no functional impairment (Level 1, score = 0) to the highest level of functional impairment (Level 6, scores = 26–33) as was previously described by Martyr et al. (2019). Cognitive function was assessed with the Addenbrooke’s Cognitive Examination-III (ACE-III; Hsieh et al., 2013), which has a maximum possible total score of 100 with higher scores indicating better cognitive function.

### Procedure

Information was collected from people with dementia and informants who were visited at home by a researcher on three occasions spread over a few weeks. Informed consent was obtained from both the person with dementia and from the informant (where available).

### Data Analysis

Data were analyzed using IBM SPSS v25. The number of participants varies by analyses due to variations in the level of missing data, with sample size numbers indicated for each analysis in the results. Associations between key characteristics (age, gender, type of dementia, functional ability, living situation, and general cognitive function) and the outcomes of interest (physical activity, loneliness, depression, and QoL) were assessed with *t* tests, chi-square, Spearman’s rho correlations or analysis of variance (ANOVA) as appropriate to the type of data, conditional upon test assumptions being met. As previous AAI research has focused on dogs, this study considered first pet ownership in general (i.e., any animal), and second dog ownership specifically, before considering the interactions of pet and dog ownership with involvement in the care of the pet as potential predictors. Multiple generalized linear and binary regressions were then conducted, adjusting for key characteristics that were significantly associated with each outcome. Separate regressions were conducted for each key predictor: pet versus no pet, dog versus no dog, having a pet and involvement in its care or having a pet and no involvement in its care versus no pet, and finally having a dog and involvement in its care or having a dog and no involvement in its care versus no dog. Holm–Bonferroni corrections for multiple comparisons were applied to all analyses.

## Results

Of the 1,547 people with dementia in the baseline IDEAL cohort 1,542 answered the pet questions; however, not all participants had data for all other included variables. The majority of the participants (*n* = 1,075; 69.7%) responded that they did not have any pets, while the remaining 467 (30.3%) participants responded that they had at least one pet. The most prevalent type of animal was a dog (*n* = 271, 17.5%) followed by a cat (*n* = 197, 12.7%), with 80 participants (5.2%) having a different type of animal, as well as or instead of a dog or cat. Other kinds of animal were horses (*n* = 5), fish (*n* = 22), birds (*n* = 39), guinea pigs (*n* = 4), tortoises (*n* = 4), hamsters (*n* = 1), rabbits (*n* = 3), ferrets (*n* = 1), and various reptiles (*n* = 6). Of those with pets, 330 (70.7% of those with pets) were involved in the care of their animal, with 195 participants (41.8% of those with pets) involved in the care of a dog specifically. Table 1 provides an overview of sample characteristics and key variables including the number of participants with data for these variables and whether there were differences between those who did and did not have a pet. Table 1 shows that there were unadjusted significant associations between those with and without a pet for age, living situation, physical activity, cognitive function, and depression; the unadjusted associations between pet ownership and gender, type of dementia, functional ability, loneliness, and QoL were nonsignificant. In the unadjusted associations, pet owners were significantly more likely to walk 3 hr or more per week, to be younger, to live with a spouse or other, to have better cognitive function, and to be depressed than non-pet owners.

**Table 1.** Descriptive Information for Key Variables and Their Association With Pet Ownership.

Variable (*n*)	Categories/range	*n* (%)/mean (*SD*)	Yes pet (any animal)	No pet	Test statistic
Age (1,542)	43–98	76.35 (8.60)	73.01 (9.20)	77.80 (7.84)	10.43***(*t*)
Gender (1,542)	Male	866 (56.2%)	278 (59.5%)	588 (54.7%)	3.09 (χ^2^)
	Female	676 (43.8%)	189 (40.5%)	487 (45.3%)	
Type of dementia (1,542)	AD	856 (55.5%)	240 (51.4%)	616 (57.3%)	4.61*(χ^2^)
	Non-AD dementia	686 (44.5%)	227 (48.6%)	459 (42.7%)	
Functional ability (1,489)	Level 1	136 (9.1%)	35 (7.7%)	101 (9.7%)	
	Level 2	422 (28.3%)	123 (27.2%)	299 (28.8%)	
	Level 3	286 (19.2%)	87 (19.2%)	199 (19.2%)	4.98 (χ^2^)
	Level 4	396 (26.6%)	119 (26.3%)	277 (26.7%)	
	Level 5	187 (12.6%)	68 (15.0%)	119 (11.5%)	
	Level 6	62 (4.2%)	20 (4.4%)	42 (4.1%)	
Living situation (1,536)	Alone	285 (18.6%)	63 (13.5%)	222 (20.7%)	
	With spouse/partner	1,163 (75.7%)	355 (76.3%)	808 (75.4%)	31.36***(χ^2^)
	With Other	88 (5.7%)	47 (10.1%)	41 (3.8%)	
Cognitive function (1,440)	21–99	69.28 (13.19)	71.06 (13.19)	68.53 (13.19)	3.35* (*t*)
Physical activity (1,505)	3 hr or more per week	684 (45.4%)	246 (53.6%)	438 (41.9%)	17.68*** (χ^2^)
	Less than 3 hr per week	821 (54.6%)	213 (46.4%)	608 (58.1%)	
Loneliness (1,441)	Not lonely	934 (64.8%)	282 (64.1%)	652 (65.1%)	0.15 (χ^2^)
	Lonely	507 (35.9%)	158 (35.9%)	349 (34.9%)	
Depression (1,497)	Not depressed	1,043 (69.7%)	296 (65.5%)	747 (71.5%)	5.37*(χ^2^)
	Depressed	454 (30.3%)	156 (34.5%)	298 (28.5%)	
Quality of life (1,402)	17–52	37.00 (5.92)	36.29 (5.75)	37.00 (6.28)	2.01*(*t*)

*Note.* Complete data *n* represents number of participants that had complete data for that specific variable and for the pets variable. AD = Alzheimer’s disease.

* *p* < .05, ***p < .001, bold indicates significant at the 5% level after Holm–Bonferroni correction.

A series of adjusted regression analyses was used to assess the associations of pet ownership, dog ownership, and the interaction with caring for the animal with physical activity, loneliness, depression, and QoL (see Table 2). All models were adjusted for those variables that were significantly associated with the outcomes; the associated covariates are discussed in relation to each outcome below.

**Table 2.** Multiple Linear and Binary Regressions for the Associations of Pet Ownership and Pet Care With Loneliness, Walking, Depression, and Quality of Life.

Pet status		Physical activity (less than 3 hr per week ref) OR (95% CI)	Lonely (not lonely ref) OR (95% CI)	Depression (not depressed ref) OR (95% CI)	Quality of life (linear) change in score (95% CI)
Pets	Yes, has a pet	1.42** [1.10, 1.84]	0.93 [0.72, 1.20]	1.23 [0.95, 1.58]	−0.02 [−0.69, 0.65]
	No pet	Ref	Ref	Ref	Ref
Dog	Yes, dog	1.83*** [1.35, 2.49]	0.86 [0.63, 1.17]	1.27 [0.95, 1.72]	−0.35 [−1.15, 0.45]
	No dog	Ref	Ref	Ref	Ref
Pet*Care	Pet and cares	1.80*** [1.35, 2.40]	0.77 [0.58, 1.04]	1.03 [0.77, 1.38]	0.63 [−0.12, 1.38]
	Pet and no care	0.76 [0.50, 1.17]	1.38 [0.92, 2.01]	1.79** [1.21, 2.68]	–1.58** [−2.67, 0.50]
	No Pet	Ref	Ref	Ref	Ref
Dog*Care	Dog and care	2.46*** [1.72, 3.53]	0.65* [0.45, 0.93]	1.05 [0.68, 1.33]	0.49 [−0.15, 2.01]
	Dog and no care	0.87 [0.50, 1.53]	1.66 [0.99, 2.80]	2.21** [1.32, 3.69]	–2.13** [−3.55, 0.72]
	No dog	Ref	Ref	Ref	−0.50 [−1.41, 0.42]

*Note.* Physical activity models adjusted for age, gender, AD vs. non-AD, functional ability and cognitive function; loneliness models adjusted for age and functional ability; depression models adjusted for AD vs. non-AD and functional ability; quality of life models adjusted for age, AD vs. non-AD, functional ability, and cognitive function. Ref: reference group. OR = odd ratio; CI = confidence interval; AD = Alzheimer’s disease.

*p < .05, **p < .01, ***p < .001, bold indicates significant at the 5% level after Holm–Bonferroni correction.

### Physical Activity

Age, gender, type of dementia, functional ability, living situation, and cognitive function were significantly associated with physical activity and were adjusted for in the following analyses. Those with pets were 1.4 times more likely to walk for over 3 hr per week compared with those without a pet (*N* = 1,361, *p* = .006). Those with a dog were 1.8 times more likely to walk for over 3 hr per week compared with those without a dog (*N* = 1,360, *p* < .001). Those with a pet who were involved in its care were 1.8 times more likely to walk for over 3 hr per week compared with those without a pet (*N* = 1,358, *p* < .001). Those with a dog who were involved in its care were 2.5 times more likely to walk for over 3 hr per week in comparison with those without a dog (*N* = 1,357, *p* < .001). There was no difference in the amount of walking between those with a pet or dog with no involvement in the animal’s care and those with no pet or dog.

### Loneliness

Age, living situation, and functional ability were significantly associated with loneliness and were adjusted for in the following analyses. Those who had a dog and were involved in its care were 35% less likely to be lonely than those who had no dog (*N* = 1,397, *p* = .018). There was no difference in loneliness in the adjusted models between those with and without a pet, those with a dog, or those with no involvement in the pet’s care.

### Depression

Type of dementia and functional ability were significantly associated with depression and were adjusted for in the following analyses. Those with a pet who were not involved in its care were 1.8 times more likely to be depressed than those with no pet (*N* = 1,445, *p* = .004). Similarly, those with a dog who were not involved in the dog’s care were 2.2 times more likely to be depressed than those with no dog (*N* = 1,444, *p* = .003). There was no difference in depression levels in the adjusted models between those with and without a pet, those with a dog, or those involved in the pet’s care.

### QoL

Age, type of dementia, and functional ability were significantly associated with QoL and were adjusted for in the following analyses. Having a pet but no involvement in its care was associated with a 1.58 point decrease in QoL score in comparison with having no pet (*N* = 1,357, *p* = .004). Similarly, having a dog but with no involvement in the dog’s care was associated with a 2.13 point decrease in QoL score in comparison with having no dog (*N* = 1,356, *p* = .003). There was no significant difference in QoL scores in the adjusted models for those with or without a pet, those with a dog, or those involved in the pet’s care.

## Discussion

This is the first study to our knowledge that has quantitatively investigated associations of self-reported pet ownership and pet care with loneliness, physical activity, depression, and QoL in a large cohort of people living with dementia. The results indicate that the associations are complex and are likely to be influenced by more than just pet ownership; the involvement of the person with dementia in the care of the animal was a key factor in the associations. After adjustment for covariates, physical activity was the only outcome associated with pet ownership without considering involvement in care for the pet. Those who had a pet of any kind and specifically a dog were significantly more likely to walk over 3 hr per week than those with no animal; the associations were stronger when care for the animal was included, suggesting that being actively involved in caring for an animal is an important aspect of the benefits of pet ownership for people living with dementia. In regards to loneliness, the only significant association was for those with a dog and who were involved in its care; this group were significantly less likely to be lonely than those with no pet. In contrast, the only significant associations for depression and QoL were in those with a pet, or specifically a dog, and were not involved in its care; this group were significantly more likely to have depression and lower QoL scores than those with no pet. This finding and the lack of direct associations between pet ownership on its own and depression or QoL suggests there may be something specific about having involvement in the care of the animal.

Previous research conducted with the wider population has generally found more positive and direct physical and psychological outcomes associated with pet ownership than were found here (e.g., Barker & Wolen, 2008; Beetz et al., 2012; Curl et al., 2016; Dall et al., 2017; Hughes et al., 2020; Hui Gan et al., 2019; Krause-Parello, 2012; Obradović et al., 2019). This is the first study to look at the role of pet ownership from the perspective of people with dementia and so it is possible that there may be more challenges associated with pet ownership for this group; for instance, people may become less physically able to care for a pet, and there is a potential increased risk of falls that could be associated with having certain types of pet in the home (Kurrle et al., 2004; Obradović et al., 2019). This could explain why some quantitative studies have found more negative than positive outcomes associated with pet ownership in older populations (Obradović et al., 2019; Parslow et al., 2005). Instead, qualitative studies have generally reported more favorable outcomes for older pet owners, such as reduced loneliness and increased socialization through providing comfort and safety, purposeful routine and structure, and a meaningful role, focus, and diversion (Connell et al., 2007; Hui Gan et al., 2019). The current study is the first to show both positive and negative outcomes and highlight the variables that may contribute to this variation.

There may be a number of other variables not considered here that explain the association between having a pet but no involvement in its care and a significantly higher likelihood of having depression or reduced QoL. The analyses for these outcomes were adjusted for functional ability so there may be more to the association than whether or not the person is able to help with the care of a pet. It is possible that “depressive realism” (Alloy & Abramson, 1979) may be a factor, with those who feel more depressed having less involvement in the care of the animal. In addition, the carer’s approach to and experience of caring has previously been associated with QoL and wellbeing in people with dementia (Kim & Park, 2017; Quinn et al., 2020). It could be the case that carers employing an “enabling” approach to care are also more likely to support the person with dementia to continue to be involved in pet care. Both the approach to care and the involvement in caring for the animal may therefore contribute to lower loneliness and depression, and better QoL.

There may be a complex interplay between the positive and negative aspects of pet ownership described above, which could result in minimal associations in such a large sample as this cohort study. In addition, there are some limitations to this study that should be considered. As the IDEAL programme has a wide scope, to avoid over-burdening participants and due to practical constraints, only a limited number of questions relating to pet ownership were included. For example, the physical activity question was limited to walking, whereas pets may also reduce other nonsedentary activity. There are also other aspects of pet ownership that are likely to be important, such as the amount of time the person with dementia spends interacting with the pet, which were not investigated as part of this study; it should be noted that caring for a pet is different from the bond that a person has with his or her pet, a person with dementia may have a strong bond with his or her pet but be unable to care for it. Moreover, we cannot infer the direction of any associations. It is possible that those who walk more are more likely to get a pet and specifically a dog, or people may get a pet in response to their depression or loneliness (e.g., Pikhartova et al., 2014). Future longitudinal work could help to identify the direction of the associations and potential explanations for these. However, the level of pet ownership identified in this large cohort study of community-dwelling people with dementia suggests that pet owners with dementia are an important population in their own right. Identifying where the benefits and difficulties of pet ownership lie could help people with dementia and their carers to make difficult decisions around taking on and keeping pets. For example, the ability of the carer to facilitate interaction between the person with dementia and the pet may be important to consider.

Despite some limitations, this study represents an important first step in providing generalizable evidence as to the associations between pet ownership and physical activity, loneliness, depression, and QoL in people with dementia. Pet ownership, and specifically dog ownership, is associated with higher levels of walking, with stronger associations when the person is also involved in the care of the animal. Having a dog and being involved in its care is associated with lower likelihood of being lonely, while having a pet and not being involved in its care is associated with higher depression and lower QoL. This may indicate something distinctive about the characteristics or environments of those who have a pet but have no involvement in its care. The IDEAL programme will allow for continued follow-up of the cohort, making it possible to identify those who may most benefit from pet ownership, and any longer-term positive and negative outcomes for pet owners. Focused qualitative research is also needed to help specify the potential benefits and difficulties of having a pet for community-dwelling people with mild-to-moderate dementia.
